# A Framework for Successful Adoption of Surgical Innovation

**DOI:** 10.1177/15533506221074612

**Published:** 2022-03-22

**Authors:** Mona Seyed Esfahani, Salman Heydari Khajehpour, Gelareh Roushan-Easton, Robert D Howell

**Affiliations:** 1 6657 Bournemouth University, Poole, UK; 2 6655 University Hospitals Dorset NHS Foundation Trust, Dorset, UK

**Keywords:** innovation adoption, robotic surgery, surgical innovation, digital health

## Abstract

**Background:**

Innovation Adoption Frameworks are applied in healthcare industry, but surgical innovation does not follow the same models as medical innovation and it is not always adopted fully by members of the team.

**Purpose:**

The aim of this paper is to develop a framework for successful adoption of surgical innovation.

**Research design:**

This paper is inspired by design thinking. Based on a pragmatic research philosophy, a mixed method approach was selected including semi-structured interview and focus groups, following a questionnaire.

**Study sample:**

A sample of five specialists in the field (doctors and managers) were selected for interview. Six focus groups were conducted. On average, five people were involved in each focus groups, 30 participants in total, including consultants, senior and junior ward nurses, health care assistant (HCA), cancer nurse specialist, stoma nurses, theatre senior and junior staff.

**Data collection/analysis:**

Qualitative data was collected and analyzed using Thematic Analysis.

**Results:**

Following a design thinking approach; firstly, an initial Surgical Adoption Model was proposed, based on the existing literature. Then, the challenges, processes and teams involved in Robotic Surgery adoption, an existing surgical innovation in a local NHS hospital, were explored. Five main themes were extracted from interviews and focus groups data - *‘Innovation Perception’, ‘Guilty vs. Undervalued’, ‘Knowledge is Power’, ‘Ex-novation’* and ‘*Facilitators and Super-users*’. This resulted into the development of an adapted Surgical Innovation Framework.

**Conclusions:**

The Surgical Innovation Framework incorporated the themes extracted from the data. The framework is unique within the field of surgical innovation and is designed with the aim of improving surgical innovation adoption success rate. Future research can trial the framework to evaluate its effectiveness.

## Background

Innovation is a vital factor in the success of the Healthcare industry and is promoted heavily by the National Health Services (NHS). The NHS has a long-term plan to provide a service fit for the future and has been a major investor in science and technology supporting innovation for NHS England. Innovation is addressed by the NHS as a crucial element which ‘help(s) to prevent diseases, speed up diagnosis, improve safety and efficiency of services and increase patient participation in decision making, self-management and research. This will lead to better health outcomes and a more sustainable NHS’.^1^

However, innovation has not always been successfully adopted and implemented within the NHS institutions. A report, funded by Academic Health Science Networks (AHSN) and published by Kings Fund, explained the slow adoption of innovation in the NHS. One factor highlighted was the lack of funding and the fact that from the limited budget available, there is near to none allocated for staff adoption and spread of innovation in NHS.^2^ National Health Services England published a figure of £1.2 billion annual spending on research and development, but only a £50 million annual spending to support innovation and dissemination (NHS England). Dissemination financial support refers to the proportion of investment spent on human knowledge, skills, and training in the adoption of innovation that determines the innovation’s level of success or failure. This is directly aligned with the challenge identified in this research; that is, the knowledge and training needed for the successful adoption and diffusion of a surgical innovation.

Castle-Clarke et al. reporting on why the NHS is struggling, explains how innovation is seen as a luxury ‘to be attempted when everything else is going well, rather than as a core part of improving quality and efficiency’^3(p6)^. Looking into Medtech innovations, they further identified procedural factors contributing to the slow adoption of innovations such as budgeting, taking a supply-focused approach, having unclear innovation management roles, and lacking strong adaptive leadership and change management procedures. These factors fit well with innovation adoption frameworks^4,5^ and models implemented within healthcare industry.^6^ However, surgical innovation adoption is not explored as detail.^7^

Medtech innovations are commonly addressed in innovation adoption frameworks within health industry. Medtech innovations include disposables, capital equipment and surgical procedure innovations. Surgical innovation is defined as ‘a new or modified surgical procedure that differs from currently accepted local practice, the outcomes of which have not been described, and which may entail risk to the patient’^8(p1206)^. It is adopted across sub-specialities in order to improve patient outcome.^9^ Adoption occurs when there is an increase in the ‘number of overall surgeons doing the procedure over time, which will occur until it is either accepted by surgeons or discarded’^10(p1092)^. The UK has been at the forefront of surgical innovation, however, some surgical innovation that was discovered and developed in the UK, has been adopted more rapidly elsewhere, resulting in UK patients being the last to benefit from these surgical innovations.^11^

Many scholars have considered adoption as a decision-making process.^6,12,13^ Wisdom et al. for instance explained Medtech adoption as ‘the decision to proceed with a full or partial implementation of an evidence-based practice’^13(p480)^. In this view, adoption starts with an awareness of innovation or pre-adoption, followed by peri-adoption which refers to the continuous access to innovation information, and finally established adoption where the adopter commits to the adoption decision.^6^ Some academics have considered the organizational aspect of Medtech adoption. For example, Frambach and Schillewaert^14^ explain a two-stage process of (i) making an organizational decision to follow the adoption and (ii) the staff acceptance of the innovation. In this model, adoption either results in implementation or de-adoption. Based on the literature, for many adoption frameworks, and in particular the ones following a decision-making model, implementation or de-adoption is the final stage.^12-15^ It is argued that focussing on the implementation of innovation might result in overlooking the complex process of adoption^13^; this seems to also be one of the main factors contributing to the slow adoption of innovation within the NHS, as the dissemination and support of adoption are not prioritized.^2^

## Surgical Innovation Framework – Initiation Phase

In order to have a broad overview of the suitable frameworks, a comprehensive literature review was conducted. The authors looked into frameworks, models and systematic reviews relevant to the field of surgical and medical innovation adoption, alongside classical reputable innovation adoption models such as Rogers.^3^ Two systematic reviews of the innovation adoption literature by Greenhalgh et al^6^ and Wisdom et al^13^ were also explored. Greenhalgh et al^6^ looked extensively into literature relevant to the spread and sustenance of innovation in health service organizations. They looked into both content and process of adoption, developing an evidence-based model of innovation diffusion. Wisdom et al^13^ conducted a review on innovation adoption theories and constructs to be adapted for evidence-based innovation. Twenty theoretical frameworks were identified in this study, equally grouped into theories with a mere focus on the adoption process, and theories which ‘address adoption within the context of implementation, diffusion, dissemination and/or sustainability’^13(p480)^.

Wisdom et al^13^ highlighted the fact that diffusion literature is heavily focused on the implementation phase of the process, with less focus on exploration/adoption (pre-implementation) or maintenance/sustenance (post-implementation) phases. They further argue that literature suggests a need for understanding adoption as an interactive, multi-level entity, rather than a standalone. As such, they developed a middle-range theory integrating existing adoption theories and mechanisms, in order to improve transferability, generalizability and external validity of the adoption theories.^13^ The present study also explored various models and frameworks such as The Unified Theory of Acceptance and Use of Technology (UTAUT),^16^ Full Contingency Model of Innovation Adoption,^17^ Practical, Robust Implementation and Sustainability model (PRISM),^18^ Reach, Effectiveness, Adoption, Implementation and Maintenance,^19,20^ Theory of Reasoned Action (TRA) and Theory of Planned Behaviour (TBP)^21^ Evidence-Based Model for Diffusion of Innovations in Health Service Organizations,^6^ Framework of Dissemination in Healthcare Intervention research,^12^ Diffusion of Innovation Model^4^ and Precaution Adoption Process Model,^22^ which was implemented in the area of Clinical Practice Guidelines for Long Term Care. However not all are aligned with the focus of this study; that of a health-related innovative product and not services, and the process of initiation and adoption, rather than development.

**Figure 1.**
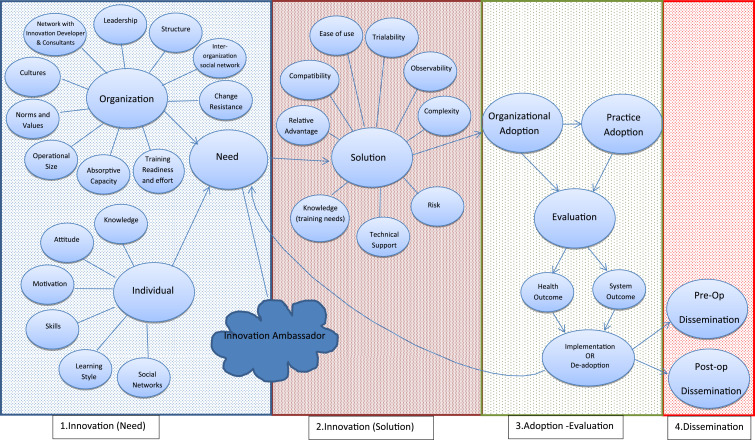
Surgical Innovation adoption Framework.

As a result of the literature review, models fitting with surgical innovation, were identified as follows: the Evidence-Based Model for Diffusion of Innovations in Health Service Organizations,^4^ the Framework of Dissemination in healthcare intervention research,^12^ Practical, Robust, Implementation and Sustainability model,^18^ Barkun et al.’s surgical evaluation paper^10^ and Wisdom et al.’s review.^13^ Considering the above models and studies, the initial framework was formed (Figure 1). Four main sections were designed as

(1.) Innovation (Need)
(2.) Innovation (Solution)
(3.) Adoption and Evaluation
(4.) Dissemination

Factors were also added based on recent reports on undermining the dissemination and support of innovation adoption within the NHS.^3^ Innovation development stages were not considered in this framework; a working assumption that the surgical innovation is ready to be introduced as a quality improvement practice was made, given the complicated nature of surgical innovation introduction, which is still inconsistent and self-regulated.^10,23^ During the primary research, the initial Surgical Innovation Framework (SIF) was explored, and all of the factors are discussed with health professionals.

## Method

**Figure 2.**
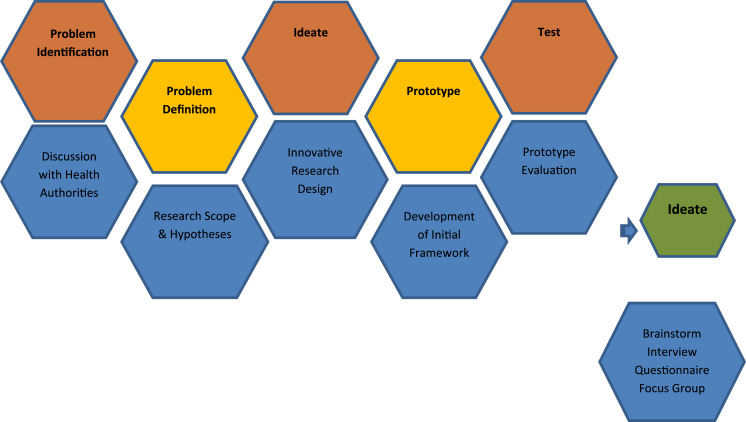
Design thinking process.

This paper is inspired by design thinking (Figure 2); as methods associated with it proved to be beneficial in innovation development.^24^ Design thinking is used in the innovation context, to help business and industry to understand disruption, to sustain competitiveness and to power strategic innovation.^25^ This study aimed to solve a problem identified by the surgical department of a local hospital, with the end goal of introducing a human-centred solution. Hence, design thinking was adopted as a systematic way to organize the research process.

**Figure 3.**
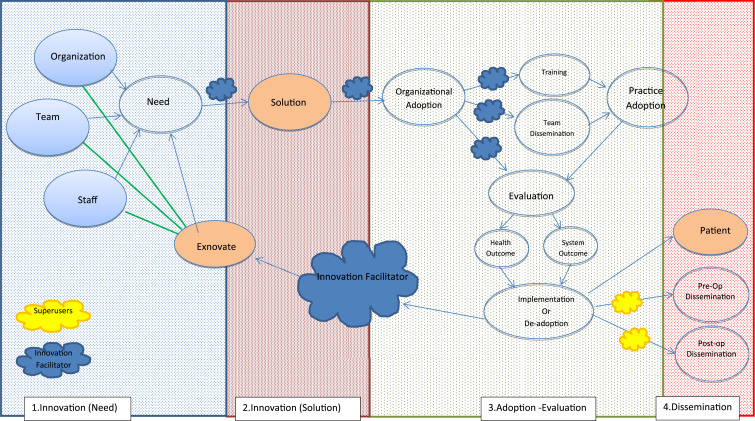
Adapted Surgical Innovation Framework (SIF).

*Problem Identification*: challenges faced in adoption of Robotic Surgery as an innovative Surgical tool; *Problem Definition*: literature review was conducted to identify the existing Medical Innovation adoption frameworks and what is missing and initial SIF is designed; *Ideate*: the most suitable research design, containing an inductive and deductive approach and qualitative methodology was identified; *Prototype*: the initial SIF designed based on the literature and *Test*: the initial SIF was tested using Robotic Surgery as a surgical innovation. In this stage, based on the pragmatic research philosophy, a mixed method approach was selected including semi-structured interview and focus groups, following a questionnaire. The result of the Test Step was an adapted SIF it fits with the structure and requirements of the hospital, addresses the gaps identified in the initial SIF (Figure 3); and enables the study to address the identified problem.

Prior to the implementation of research, the study received ethical approval from IRAS and Bournemouth University. A sample of five specialists in the field (doctors and managers) was selected for interview. A semi-structured interview guide was designed, extracted from the initial SIF. The candidates for the interview included the Director of Operation for Surgery, two Colorectal Consultants (including the clinical director for surgery) involved in performing Robotic Surgery, and two anaesthetic consultants that were directly involved in pre-assessment and anaesthetising the patients. Each interview was between 60 to 90 minutes.

For the focus groups, an invitation was sent out to the selected health care professionals that were involved in the patient’s care path. 30 participants agreed to take part in the focus groups. A semi-structured focus group guide was designed based on the initial SIF. Six focus groups were conducted. On average, five people were involved in each focus group, including consultants, senior and junior ward nurses, health care assistant, cancer nurse specialist, stoma nurses and theatre senior and junior staff. Focus groups were moderated by at least one academic and a surgeon, who was familiar with Robotic Surgery. The presence of the surgeon resulted in a more comfortable environment for staff to discuss and put forward their opinions and concerns. Furthermore, it helped the research team to understand terminologies and hospital policies and procedures better.

Candidates in the interview and participants in focus groups were given an information sheet and signed a consent form. Sessions were recorded using professional recording equipment and the data was kept confidential. The data was analysed and coded following a thematic analysis methodology. Thematic analysis is widely used in analysing qualitative data^26^ for identifying and reporting patterns in the data. Nvivo was used which helped with some steps of thematic analysis and provided researchers with quantitative insight.

## Results

The development of an adapted SIF was based on two testing stages. First, SIF discipline expert feedback and second, Application. In first stage, main comments were on a need to add ‘team’ and dividing the unit of individual to ‘patient’ and ‘staff’. There also needed more clarity on Organizational adoption and Practice adoption. In stag 2, Application, the primary data from interviews and focus groups were analysed, the adapted SIF was developed (Figure 3) and six main themes were emerged from the data.

## Innovation Perception

Innovation was perceived as a ‘new ides’, ‘a solution’ or ‘improvement or modification of an existing service or product’, which is linked well with classical definition of innovation. However, innovation was also perceived as luxury and unnecessary. Comments such as ‘*is it worth going through the pain?’ or ‘If it’s not broken, why fix it?’,* which indicated how superfluous it can be perceived, citing reasons such as its complexity, resources limitation, intangible nature of some innovation, the top-down decision-making system and the system’s resistance to change. This is in line with previous literature, for instance how intangible innovation is not included in measurement models of company innovativeness.^27,28^

There were some discussions on how *‘men like their toys’*, implying that adoption occurs for the sake of personal achievement. However, most participants highlighted the importance of patient safety and staff experience, even if it meant staff going through extended training.

*‘It is a lot of set up and takes a lot of extra time, and some of it is quite complicated but my impression overall, it’s better for patient.’*

A point raised by mainly nursing and care team was that some innovation has taken away the human connection element of their job which changes their attitude towards innovation. The de-humanizing effect of technology is evident when looking into social relations and technology.^29^

*‘People don’t look at the patient anymore; they’ll look at the pad and figure out what’s going on.’*

While discussing Robotic Surgery, participants explained the need for a surgical innovation to be patient-centred. Although some participants understood the patient-centred nature of Robotic surgery even if it is an indirect effect through staff quality of practice, the link was not always clear for everyone.

*‘Sometimes you improve things for the patient in slightly different ways, so actually if I make the life of a surgeon easier, then that will ultimately benefit the patients, so making the life of a surgeon may not be an immediate direct benefit’.*

However, there were many discussions around the NHS needing to change the over-emphasis of patient safety and evidence-based studies, and the need to speed up innovation acceptance.

*‘We are never going to innovate if we are just waiting for someone else to prove that something works perfectly, and is absolutely brilliant and just wait and wait until it is absolutely proven before we adopt it’.*

To reflect this theme, the adapted SIF included an Innovation Facilitator role in identifying the need (problem); to ask for staff opinion on existing issues and challenges, and recognizing the solution (innovation); to introduce the most suitable innovation available to tackle the problem, explain the innovation to the staff to clarify the patient-centred nature of the innovation and how the product/service can address the problem.

## Guilty vs Undervalued

The most surprising theme was the emotional challenges staff faced during the innovation adoption process. This was discovered during focus groups where junior staff explained how they felt undervalued, not part of the team and excluded when they were made aware of an innovation, post-adoption. Junior staff also felt they were not heard if they raise any concerns around an existing innovation or an idea.

*‘We’re told what to do, we’re told what is our responsibility, we’re told we’ll be disciplined if we don’t do that, but we are not encouraged to understand why we have to do…[and]…actually we live in a democracy and we have freedom of speech, but it doesn’t feel like that when you’re at work’.*

Junior staff felt guilty discussing their feelings; but felt it necessary for senior staff to hear it.

‘P1: God I sound *a bit moany don’t I?* P2: *No you don’t no*. P1: *I think it’s useful for them to hear that’*.

Senior staff felt guilty and shocked upon learning this insight and responsible to have had informed the junior staff.

*‘I was feeling very guilty and I was thinking well maybe we should have got the ward on board when we were going and I thought well maybe was it for me to think about that…’*.

However upon being informed about an existing *Innovation Facilitator*, senior staff expressed how this could have been avoided if the *Innovation Facilitator* was involved in the process and took responsibility.

*‘But actually, this is the Innovation Officer’s responsibility, if the Innovation Officer did a course or spoke to (the rep) and got some training, he could have gone and trained the ward staff’.*

The importance of recognizing staff emotional labour is highlighted in academic literature,^30,31^ so in adapted SIF, *Innovation Officer* is responsible to inform all the staff involved (direct and indirectly) of the innovation and facilitate any training necessary.

## Knowledge Is Power

*‘If something is meaningful to me and it makes sense to me, then I absolutely will do that sincerely’.*

Apart from surgeons who were working with the Robotic device, people not directly involved with the innovation were mostly unaware of Robotic Surgery. This lack of knowledge was discussed at all stages of SIF. At stage 1 (initiation), staff did not have enough knowledge of the process, or know who to approach for their ideas and opinions; furthermore, staff were not encouraged to initiate innovation. In stage 2 (solution), staff were unaware of the existing innovative products and services, hence could not come up with an existing solution for the problem they identified in Stage 1. In Stage 3 (adoption and evaluation), the lack of knowledge led to a lack of interest, higher perceived complexity and a lack of trust, hence lower adoption. Finally, in Stage 4 (dissemination), if anything, the need for knowledge was more apparent in order to address the issues associated with themes 1 and 2. This is in line with academic literature on the positive effect knowledge has on attitude and decision-making such as adoption.^4,32^ Knowledge dissemination and acquisition is included in the adapted SIF via training, and as part of the Innovation Facilitator and Super-user roles.

## Ex-novating

*‘We are innovating, but what you should also be doing is ex-novating and taking the stuff out and we are bad at taking stuff out’.*

Looking back at the conversations with participants, the sentiments of *‘If it is not broken, why fix it’,* and *‘innovation is a luxury’* were highly present. If hospitals do not ex-novate and take out the products and services which are out-of-date, cannot be updated or no longer productive, then the need for bringing innovation is not going to be there.

*‘The reasons you’re not ex-novating is because the people that see you need to be ex-novating, don’t have a voice or don’t think they have a voice or we are not giving them a voice, we are not giving them the forum to actually sort of tell us’.*

This links strongly with themes 1 and 2; the hospital needs to tackle the knowledge and emotional connection with staff in order to make ex-novating happen. Participants expressed how some of the existing innovations were not perceived; they complained about the hospital not monitoring the innovation’s efficiency so that if it does not work, then should be replaced. Monitoring and evaluating are encouraging factors for ex-novating. An example is the conversation below:

*‘P1: …we had a new machine – the bed scales – so we could weigh people that were in the bed that we couldn’t get out of the bed, or hoist away because they were just too unwell and we had training on that. So everyone on the ward was invited and the trainer would come on the ward like every day and we would have a list and whoever had had the training, you had to sign your name and then we were all shown how to use the bed scales’ P2: ‘I wasn’t’ P1: ‘ …and then they broke [laughs] and they can’t use them. Were you not?’ P2: ‘No I wasn’t shown how to use them by anyone.’* ***…*** *P3: ‘Yeah then they broke.’ P1: ‘Yeah it’s now covered with like a plastic…’ P2: ‘Yeah has been for four months.’ P3: ‘We spent so much money on it’.*

Ex-novating is regarded as in important, yet undermined factor in innovation initiation and adoption; based on literature, more attention is required to understand and include ex-novation in innovation management process.^33-35^ Ex-novation is incorporated in the adapted SIF; it needs to be facilitated by the Innovation facilitator as one of the main factors impacting innovation initiation and the cycle of adoption. Also, in order to ex-novate, there is a need to evaluate the innovation’s productivity by approaching the teams involved in the adoption process from health and system (organization) points of view.

## Facilitator and Super-users

All the themes above are strongly linked to a need for innovation facilitation in the hospital. Although an innovation facilitator role currently exists at Royal Bournemouth Hospital, all but the two senior staffs (with managerial roles) knew about the role. The was raised repeatedly,

*‘I have worked in (the hospital) since it opened and I have never heard of an Innovation Officer.’*

There were feelings of anger and disappointment of not knowing about the Innovation Facilitator. There were discussions on the necessity of such a role to facilitate, co-ordinate, train, inform, disseminate, plan and do networking activities to name a few. Furthermore, the role of a ‘super user’ or ‘champion’ in each department was suggested by participants. Staff had a good experience with champions in wards, for process and service.

*‘So the super user is like the champion, so they are going to be trained first’*

*‘Yeah then look at champions because you’ve got champions for certain things, so for training on the wards we have a blood group, a diabetes champion, manual handling, end of life champion, you know, so they are your go to if you’ve got any questions…’*

*‘They did that with the new air mattresses, the hybrids. They sent the reps out to go through everything and then each ward has got a specific champion.’*

**Figure 4.**
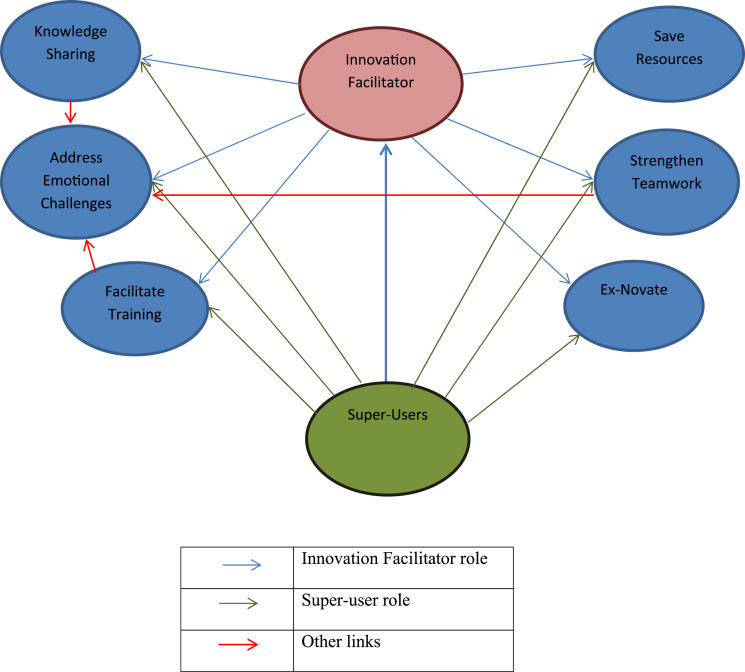
Role of Innovation Facilitator and super user.

To conclude, the Innovation Facilitator role/s need to be clearly defined as an organizational role and introduced to the staff, which is deemed important but a shortfall based on academic research.^36,37^ Super Users or Champions is a common term in care teams and have been identified as an important factor in implementation of innovation.^38^ Hence, Innovation Facilitator and Super User is included in the adapted SIF (more detail in Figure 4).

## Discussion

The study addressed the challenges faced in adoption of Surgical Innovation. Based on the results, five themes were identified and incorporated into the initial SIF, *Innovation Perception, Guilty vs. Undervalued, Knowledge is power, Ex-novating* and *Facilitator and Super Users*. Although surgical innovation was perceived in line with innovation literature, but it was also perceived as luxury and unnecessary, which was highlighted by scholars.^2,3,39^ As explained in the results, the adapted SIF addresses this by including links between Innovation Facilitator/s and the team. However, the inclusion of this role in the system might be problematic due to lack of resources. As explained in *Knowledge is Power* theme, a need for knowledge acquisition was established in the early stage of innovation initiation within initial SIF (Figure 1) and also as part of the dissemination process. Staff need to be exposed to existing innovation in order to understand what can be done and what the possibilities are and during dissemination; they also need to be made aware of the adopted innovation. The important role of knowledge is confirmed in the adapted SIF (Figure 3), which is in line with previous literature.^4,40^ During the test process, it was apparent that there needs to be a constant flow of information from the initiation stage to dissemination. Firstly, as a role for Innovation Facilitator to co-ordinate knowledge dissemination from the initiation stage (on existing innovation), the Solution stage (what innovations are available as solution to the need), the adoption and evaluation stage (training, best practice to evaluate against) and the dissemination stage (promotion and presentation). Super-users or Champions in different wards were also identified during the test process, as essential human factors. Super-users would be the link between the Innovation Facilitator and the staff, who could distribute information and make knowledge available for staff in a more specialized manner. If knowledge acquisition is encouraged and facilitated by the system, it can also address the highlighted emotional challenges staff are currently facing, as explained in *Guilty vs. Undervalued*. *Ex-novating* is also a factor resulting into a better perception of innovation, acquisition of more knowledge and a point of action on staff feedback on removing unnecessary, out of date and useless innovation.
